# Fecal Microbiota Transplantation Beneficially Regulates Intestinal Mucosal Autophagy and Alleviates Gut Barrier Injury

**DOI:** 10.1128/mSystems.00137-18

**Published:** 2018-10-09

**Authors:** Saisai Cheng, Xin Ma, Shijie Geng, Xuemei Jiang, Yuan Li, Luansha Hu, Jianrong Li, Yizhen Wang, Xinyan Han

**Affiliations:** a Key Laboratory of Animal Nutrition and Feed Science in East China, Ministry of Agriculture, College of Animal Science, Zhejiang University, Zhejiang, People’s Republic of China; b College of Veterinary Medicine, Ohio State of University, Columbus, Ohio, USA; Pacific Northwest National Laboratory

**Keywords:** autophagy, fecal microbiota transplantation, gut barrier, gut microbiota, piglets

## Abstract

Fecal microbiota transplantation (FMT) is one of the most effective ways to regulate the gut microbiota. Here, we investigated the effect of exogenous fecal microbiota on gut function from the perspective of analysis of the mucosal proteomes in a piglet model. A total of 289 differentially expressed proteins were annotated with 4,068 gene ontology (GO) function entries in the intestinal mucosa, and the levels of autophagy-related proteins in the forkhead box O (FoxO) signaling pathway were increased whereas the levels of proteins related to inflammation response were decreased in the recipient. Then, to assess the alleviation of epithelial injury in the Escherichia coli K88-infected piglets following FMT, intestinal microbiome-metabolome responses were determined. 16S rRNA gene sequencing showed that the abundances of beneficial bacteria, such as *Lactobacillus* and *Succinivibrio*, were increased whereas those of *Enterobacteriaceae* and *Proteobacteria* bacteria were decreased in the infected piglets following FMT. Metabolomic analysis revealed that levels of 58 metabolites, such as lactic acid and succinic acid, were enhanced in the intestinal lumen and that seven metabolic pathways, such as branched-chain amino acid metabolism pathways, were upregulated in the infected piglets following FMT. In concordance with the metabolome data, results of metagenomics prediction analysis also demonstrated that FMT modulated the metabolic functions of gut microbiota associated with linoleic acid metabolism. In addition, intestinal morphology was improved, a result that coincided with the decrease of intestinal permeability and the enhancement of mucins and mucosal expression of tight junction proteins in the recipient. Taken together, the results showed that FMT triggered intestinal mucosal protective autophagy and alleviated gut barrier injury through alteration of the gut microbial structure.

**IMPORTANCE** The gut microbiota plays a crucial role in human and animal health, and its disorder causes multiple diseases. Over the past decade, FMT has gained increasing attention due to the success in treating Clostridium difficile infection (CDI) and inflammatory bowel disease (IBD). Although FMT appears to be effective, how FMT functions in the recipient remains unknown. Whether FMT exerts this beneficial effect through a series of changes in the host organism caused by alteration of gut microbial structure is also not known. In the present study, newborn piglets and E. coli K88-infected piglets were selected as models to explore the interplay between host and gut microbiota following FMT. Our results showed that FMT triggered intestinal mucosal autophagy and alleviated gut barrier injury caused by E. coli K88. This report provides a theoretical basis for the use of FMT as a viable therapeutic method for gut microbial regulation.

## Abstract (precis)

The gut microbiota plays a crucial role in human and animal health, and its disorder causes multiple diseases. Over the past decade, FMT has gained increasing attention due to the success in treating Clostridium difficile infection (CDI) and inflammatory bowel disease (IBD). Although FMT appears to be effective, how FMT functions in the recipient remains unknown. Whether FMT exerts this beneficial effect through a series of changes in the host organism caused by alteration of gut microbial structure is also not known. In the present study, newborn piglets and E. coli K88-infected piglets were selected as models to explore the interplay between host and gut microbiota following FMT. Our results showed that FMT triggered intestinal mucosal autophagy and alleviated gut barrier injury caused by E. coli K88. This report provides a theoretical basis for the use of FMT as a viable therapeutic method for gut microbial regulation.

## INTRODUCTION

In recent years, the role of gut microbiota in human and animal health has gained increasing attention. A healthy gut microbiota can be considered a stable state with respect to the composition and function of the microbial community and adaption to outside perturbations (1). Increasing evidence links disruption of gut microbiota homeostasis to metabolic diseases, immune diseases, gastrointestinal diseases, and even mental diseases (2, 3). Moreover, those disruptions induce significant changes in physiological processes, such as severe intestinal inflammation, imbalance of the intestinal redox status, and the dysregulation of autophagy (4, 5). Among these processes, autophagy plays important physiological roles in host health and disease. It acts as an innate barrier to infection and plays a crucial role in recognition and degradation of intracellular pathogens (5, 6).

The intestinal epithelium has the largest mucosal surface of the body, and the production of mucins and antimicrobial proteins establishes physical and biochemical barriers to prevent enteric pathogen invasion (7). Loss of the gut barrier causes systemic immune activation, resulting in a wide range of extraintestinal autoimmune and inflammatory diseases (8, 9). Other factors, such as epithelial tight junction proteins (TJs) or the proteins involved in epithelial cell renewal, contribute to maintain an efficient gut barrier (10). All of these factors are affected by the composition and the activity of the intestinal commensal microbiota (11). The intestinal commensal microbiota also showed enhanced barrier function by driving mucosal immune homeostasis (12). Because autophagy within the intestinal epithelium plays an important role in maintaining the integrity of the intestinal barrier, defects of autophagy-related (Atg) genes increase the risk of inflammatory diseases (13, 14). Therefore, a comprehensive understanding of the barrier-assisting and immunoregulatory properties of autophagy could help to develop new strategies to prevent and treat multiple inflammatory and metabolic diseases.

Over the past decade, fecal microbiota transplantation (FMT) has drawn attention due to its success as a method of treatment in Clostridium difficile infection (CDI) patients. Studies in CDI patients revealed that diversity of gut microbiota increased following FMT, which is critical for defense against pathogens and is referred to as colonization resistance (15). A study of Parkinson’s disease (PD) mice showed that gut microbiota dysbiosis was ameliorated following FMT (16). Although FMT appears to be effective, how it functions in recipients remains poorly understood. In addition, it is unknown whether FMT exerts this beneficial effect through a series of changes in the host organism caused by alteration of gut microbial structure. Therefore, there is an urgent need to assess the evolutionary responses of gut microbiota following FMT in the context of health and disease.

Previously, we showed that exogenous fecal microbiota modulated the composition of the intestinal microbiota and enhances the expression of mucosal Toll-like receptor 2 (TLR2) and TLR4 and antimicrobial peptide β-defensin 2 in a newborn piglet model (17). On the basis of those results, we hypothesize that FMT regulates intestinal mucosal autophagy and anti-inflammatory ability. In addition, the intestinal injury caused by Escherichia coli K88 infection could be relieved by altering the composition of intestinal microbiota and its metabolites. Therefore, the aim of this study was to explore the interplay between host and gut microbiota following FMT from a multi-omics perspective through the establishment of an E. coli K88-infected piglet model. This report provides a theoretical basis for the use of FMT as a viable therapeutic method for gut microbial regulator.

## RESULTS

### Intestinal mucosal proteomes. (i) Identification and comparison of differentially expressed proteins.

In experiment I, in order to study the effects of FMT on gut function from the perspective of analysis of the mucosal proteomes, DLY (Duroc × Landrace × Yorkshire) newborn piglets were randomly divided into FMT and control groups and were inoculated orally with a fecal microbiota suspension and phosphate-buffered saline (PBS), respectively. A total of 3,815 proteins were identified with a false-discovery rate (FDR) of 1% using isobaric tags for relative and absolute quantitation (iTRAQ) analysis. A quantitative protein with a 1.2-fold change value (a ratio of >1.2 or <0.83) and a *P *value of *<*0.05 was considered a differentially expressed protein. A total of 289 proteins were found to be differentially expressed in the colonic mucosa, with 40 of these proteins being upregulated and 249 downregulated. All differentially expressed proteins are listed in Table S1 in the supplemental material. The statistical results of protein quantification, presented in volcano plot format, are shown in Fig. S1A in the supplemental material. A heat map representing data from hierarchical clustering analyses is shown in Fig. S1B.

10.1128/mSystems.00137-18.1 FIG S1 (A) Volcano plot drawn by using the fold change values and *P* values obtained by the *t* test to show significant difference between the two groups. The red spots in the figure represent the significantly differentially expressed protein; black spots represent proteins with no differential changes. (B) Heat map of hierarchical clustering analysis. Each row in the graph represents a protein (the ordinate represents a significant differentially expressed protein), each column represents a group of samples (the abscissa represents sample information), and the Log2 expression levels of differentially expressed proteins in different samples are presented in different colors in the heat map, with red representing a significantly upregulated protein, blue representing a significantly downregulated protein, and gray representing no protein quantitative information. Download FIG S1, TIF file, 1.5 MB. Copyright © 2018 Cheng et al. 2018 Cheng et al. This content is distributed under the terms of the Creative Commons Attribution 4.0 International license.

10.1128/mSystems.00137-18.6 TABLE S1 Differentially expressed proteins in the colonic mucosa. Download Table S1, XLSX file, 0.1 MB. Copyright © 2018 Cheng et al. 2018 Cheng et al. This content is distributed under the terms of the Creative Commons Attribution 4.0 International license.

The proteins that were observed to be differentially expressed between the FMT group and the control group were involved in multiple processes, such as energy production and lipid and amino acid metabolism processes. These proteins included sodium/potassium-transporting ATPase subunit beta (ATP1B), prolyl 4-hydroxylase (P4HA), phosphatidate phosphatase (PAP), facilitated glucose transporter member 4 (GLUT4/SLC2A4), and serine/threonine-protein kinase 11 (STK11/LKB1). Phosphatidate phosphatase (PAP) (EC 3.1.3.4) is a key regulatory enzyme in lipid metabolism, catalyzing the conversion of phosphatidate to diacylglycerol. Some differentially expressed proteins were also involved in other physiological processes such as autophagy, oxidative stress, and inflammatory responses. These proteins included voltage-dependent anion channel protein 1 (VDAC1), gamma-aminobutyric acid receptor-associated protein (GABARAP), tuberous sclerosis 2 (TSC2), RAS protein activator-like 3 (RASAL3), tubulin beta (TUBB), superoxide dismutase Fe-Mn family (SOD2), peroxin 16 (PEX16), nuclear factor NF-kappa-B p105 subunit (NFKB1), nuclear factor of kappa light polypeptide gene enhancer in B-cells 2 (NFKB2), and interferon (IFN) regulatory factor 3 (IRF3). Peroxin 16 (PEX16) plays an essential role in peroxisomal membrane protein targeting and *de novo* biogenesis of peroxisomes from endoplasmic reticulum.

### (ii) GO annotations of differentially expressed proteins.

**FIG 1:**
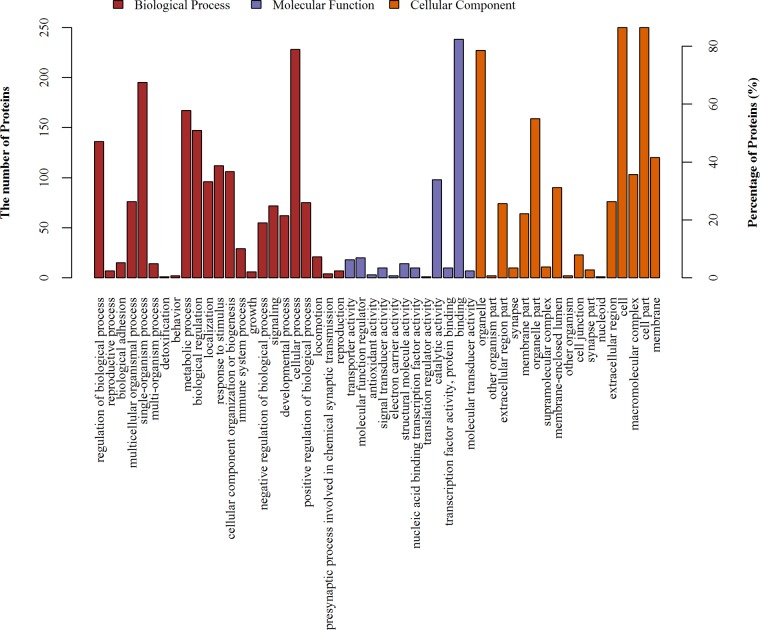
GO distribution analysis of differentially expressed proteins in the mucosa. The horizontal coordinate axis indicates the enriched GO functional classifications.

The gene ontology (GO) database is an internationally standardized gene functional classification system that was developed to comprehensively describe characteristics of different genes and their products. A total of 289 differentially expressed proteins were annotated to 4,068 GO function entries. Second-level GO terms were applied to classify proteins in terms of their involvement in three main categories (biological process, cellular component, and molecular function), and each protein was assigned at least one term. As summarized in Fig. 1, more than 87% of the differentially expressed proteins belonged to the cell compartment category, and the other two main categories of these proteins consisted of the organelle (79%) and membrane (42%) compartment proteins. The two main molecular functions of these proteins were binding (82%) and catalytic activity (34%). The top three categories of biological processes identified were cellular processes (79%), single-organism processes (67%), and metabolic processes (58%).

### (iii) KEGG pathway analysis of differentially expressed proteins.

**FIG 2:**
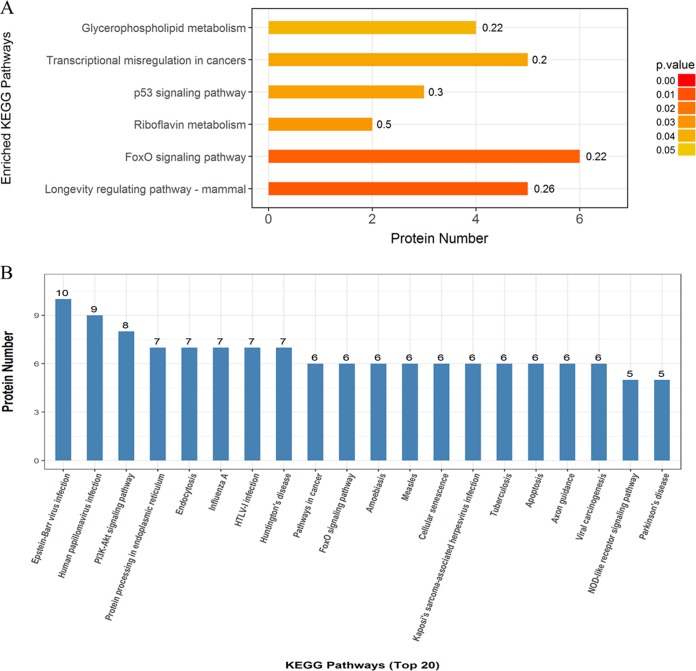
Pathway-based analysis of the differentially expressed proteins in colonic mucosa. (A) Top six pathways with significant differences. The color gradient represents the *P* values; the closer the color is to red, the smaller the *P* value is and the higher the significance level of the corresponding KEGG pathway enrichment is. (B) Top 20 pathways for enrichment of differentially expressed proteins. HTLV-1, human T-lymphotropic virus type 1.

KEGG pathway analysis was performed to identify pathways that are potentially affected by differentially expressed proteins. The top six pathways with significant differences were the longevity-regulating pathway (mammals), the forkhead box O (FoxO) signaling pathway, the riboflavin metabolism pathway, the p53 signaling pathway, the pathway of transcriptional misregulation in cancers, and the glycerophospholipid metabolism pathway (Fig. 2A). The top 20 pathways for enrichment of differentially expressed proteins are shown in Fig. 2B.

### (iv) Validation of differentially expressed proteins.

**FIG 3:**
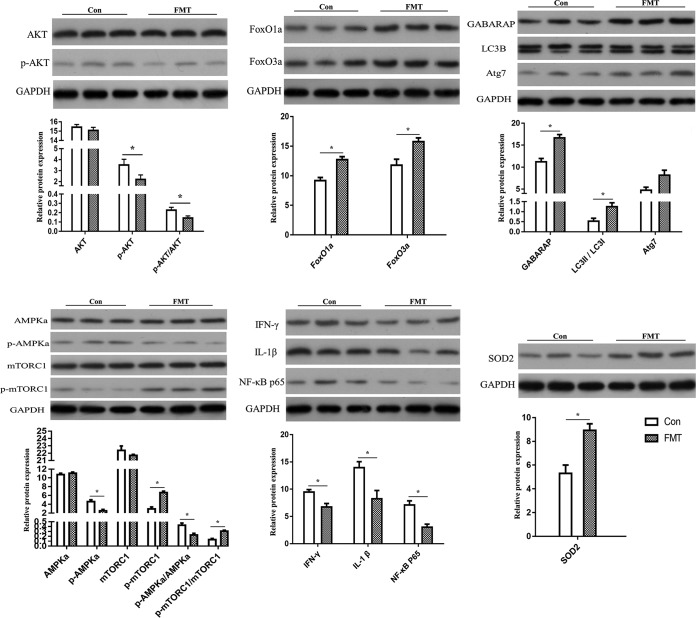
Western blot analysis of the differentially expressed proteins and selected key proteins. The results of statistical analysis are shown below the protein expression map. Values are means ± standard errors of the means. *, *P < *0.05 (*n* = 3). Con, control.

Among these differentially expressed proteins, autophagy-related protein (GABARAP), antioxidant protein (SOD2), and inflammatory response related factor protein (NF-κB p65) were selected for validation of differentially expressed proteins. Autophagy-related proteins (AKT [alpha serine/threonine kinase], FoxO1, FoxO3, LC3B, and Atg7) in the FoxO pathway, the key proteins (AMP-activated protein kinase a [AMPKa] and mammalian target of rapamycin C1 [mTORC1]) in the AMPK-mTOR pathway, and the cytokines (gamma interferon [IFN-γ] and interleukin-1β [IL-1β]) were selected as targeted proteins for further study. The results of analysis by Western blotting are shown in Fig. 3; the levels of protein expression of FoxO1a, FoxO3a, GABARAP, LC3B, Atg7, and SOD2 in the recipient mucosa were higher than those in the control. While there were no significant differences in the levels of expression of proteins total-AKT, total-AMPKa, and total-mTORC1 (*P > *0.05), the p-AKT/AKT and p-AMPKa/AMPKa expression levels were lower and the p-mTORC1/mTORC1 expression level was higher than those in the control (*P < *0.05). The protein expression levels of NF-κB p65, IFN-γ, and IL-1β in the recipient mucosa were lower than those in the control (*P < *0.05).

### Weight gain and clinical signs.

In experiment II, in order to assess the alleviative effect of FMT on epithelial injury, DLY piglets were randomly assigned to the blank group (not inoculated with bacteria) or the K88 group (inoculated with E. coli K88 bacterial suspension). The infected piglets were then inoculated orally with PBS or a fecal microbiota suspension, representing the K88-plus-PBS group and K88-plus-FMT group, respectively. In the study, E. coli K88 infection resulted in decreased weight gain and increased diarrhea incidence in piglets. However, the weight loss and diarrhea incidence of the recipient piglets were reduced following FMT. The body temperature of E. coli K88-infected piglets increased to 40.47 ± 1.03°C, while the body temperature of the recipient piglets gradually returned to normal following FMT (see Fig. S2). These results suggested that FMT relieves diarrhea caused by E. coli K88 infection and increases the weight gain of the recipient piglets.

10.1128/mSystems.00137-18.2 FIG S2 Average daily weight gain (ADG), diarrhea incidence, and body temperature of piglets. Download FIG S2, TIF file, 0.6 MB. Copyright © 2018 Cheng et al. 2018 Cheng et al. This content is distributed under the terms of the Creative Commons Attribution 4.0 International license.

### Intestinal microbiota composition and diversity.

**FIG 4:**
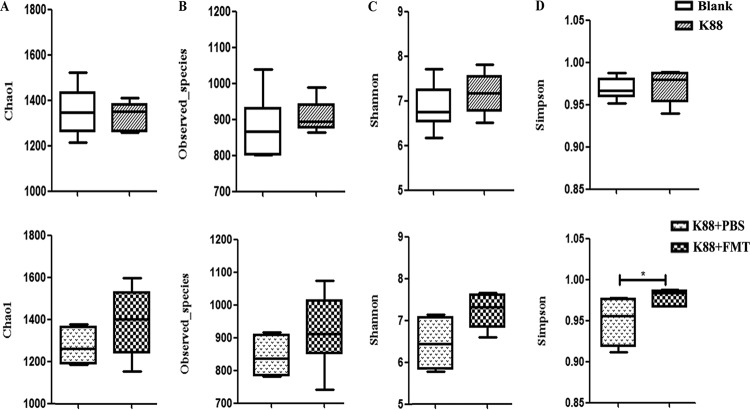
Richness and diversity of colonic microbiota. (A) Chao 1 index. (B) Observed-species. (C) Shannon index. (D) Simpson index. Values are means ± standard errors of the means. *, *P < *0.05 (*n* = 6).

**FIG 5:**
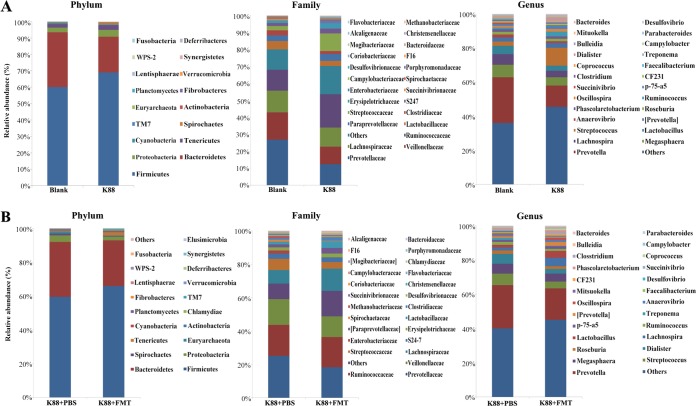
Relative abundances of colonic microbiota at the phylum, family, and genus levels. (A) Relative abundances of intestinal microbiota at three different levels in the piglets left uninfected of infected with E. coli K88 (*n* = 6). (B) Relative abundances of colonic microbiota at three different levels in the infected piglets treated with or without exogenous fecal microbiota (*n* = 6).

**FIG 6:**
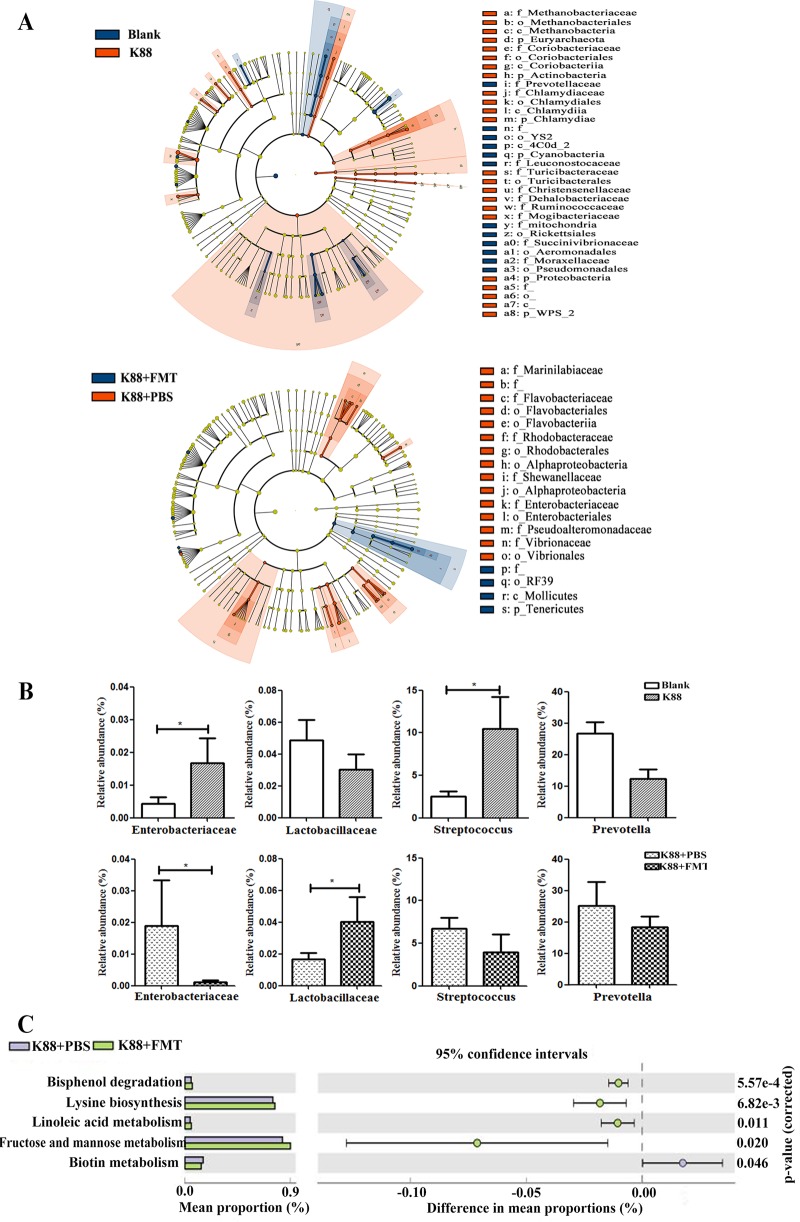
Structural changes and functional metagenomics prediction analysis of colonic microbiota. (A) Cladogram of enriched taxa based on LEfSe determinations revealing significant differences in microbial communities between the blank and K88 groups and the K88-plus-PBS and K88-plus-FMT groups (*n* = 6), respectively. Bacterial taxa with an LDA score of >2 were selected as biomarker taxa (p, phylum level; c, class level; o, order level; f, family level; g, genus level). (B) Bar graphs of the relative abundances of the members of selected bacterial families in the four groups (*n* = 6). (C) Functional metagenomics prediction of gut microbiota by PICRUSt with significant differences. The significant levels of the relative abundances are shown as error bars in the figure.

We determined the bacterial community by amplification and sequencing of the 16S rRNA gene (V3-plus-V4 region). Pretreatment statistics and quality control of microbial sequencing data are shown in Table S2. On average, 38,252 high-quality sequences were obtained per sample, with an average of 974 operational taxonomic units (OTUs) per sample in colonic microbiota. Data corresponding to the richness and diversity of intestinal microbiota in the four groups are shown in Fig. 4. There was no significant difference in Chao 1, Observed species, or Shannon and Simpson indices in piglets left uninfected or infected with E. coli K88 (*P > *0.05). However, FMT increased the alpha diversity as evidenced by the Simpson index (*P < *0.05). Relative abundances of colonic microbiota compositions with respect to the levels of phylum, family, and genus are shown in Fig. 5. *Firmicutes* and *Bacteroidetes* were the most predominant phyla in the colon of infected piglets, followed by the phyla *Proteobacteria*, *Tenericutes*, and *Spirochaetes*. The most abundant phyla were *Firmicutes*, *Bacteroidetes*, *Proteobacteria*, *Spirochaetes*, and *Tenericutes* in the recipient piglets following FMT. Linear discriminant analysis (LDA) effect size (LEfSe) determinations (Fig. 6A) (LDA score plots are shown in Fig. S3) further indicated that at the phylum level, E. coli K88 infection increased the relative abundances of *Fibrobacteres*, *Verrucomicrobia*, and *Chlamydiae* in the colon and decreased the relative abundance of *Lentisphaerae* (*P < *0.05). At the family level, E. coli K88-infected piglets had higher *Enterobacteriaceae* and *Streptococcaceae* levels and lower *Succinivibrionaceae* levels in the colon (*P < *0.05). At the genus level, *Streptococcus*, *Dialister*, and *Faecalibacterium* levels in the colon of infected piglets were increased (*P < *0.05) and a decreasing trend was found for *Succinivibrio*. The recipient piglets had lower levels of members of the phyla *Proteobacteria* and *Spirochaetes* than the infected piglets treated without exogenous fecal microbiota (*P < *0.05). Compared with infected piglets treated without exogenous fecal microbiota, levels of the family *Lactobacillus* increased significantly in the colon of the recipient (*P < *0.05), while levels of *Enterobacteriaceae* were significantly reduced (*P < *0.05). The levels of the genera *Lactobacillus*, *Succinivibrio*, *Phascolarctobacterium*, and *Parabacteroides* in the colon of the recipient were increased. In contrast, *Ruminococcus* and *Treponema* levels were decreased (*P < *0.05). E. coli K88 infection increased the relative abundances of *Enterobacteriaceae* and *Streptococcus* (*P < *0.05); when infected piglets received exogenous fecal microbiota, the relative abundance of *Enterobacteriaceae* was reduced (*P < *0.05), while the relative abundance of *Lactobacillaceae* was increased (*P < *0.05) (Fig. 6B).

10.1128/mSystems.00137-18.3 FIG S3 LDA score plot of microbial taxa with significant group differences. (A) LDA score plots of the blank and K88 groups. (B) LDA score plots of the K88-plus-FMT and K88-plus-PBS groups. Bacterial taxa with an LDA score of >2 were selected as the biomarker taxa (p, phylum level; c, class level; o, order level; f, family level; g, genus level). Download FIG S3, TIF file, 0.9 MB. Copyright © 2018 Cheng et al. 2018 Cheng et al. This content is distributed under the terms of the Creative Commons Attribution 4.0 International license.

10.1128/mSystems.00137-18.7 TABLE S2 Pretreatment statistics and quality control of microbial sequencing data. Download Table S2, DOCX file, 0.0 MB. Copyright © 2018 Cheng et al. 2018 Cheng et al. This content is distributed under the terms of the Creative Commons Attribution 4.0 International license.

We conducted a functional metagenomics prediction of intestinal microbiota using PICRUSt (phylogenetic investigation of communities by reconstruction of unobserved states) (Fig. 6C). Pathway enrichments at KEGG level 3 showed that recipient piglets had higher inferred levels of enrichment of the pathways involved in linoleic acid metabolism and in fructose and mannose metabolism as well as in biosynthesis of lysine and degradation of bisphenol (*P < *0.05). However, the level of enrichment of the pathway for biotin metabolism in the recipient was significantly decreased (*P < *0.05).

### Differential levels of metabolites and metabolic pathway.

**FIG 7:**
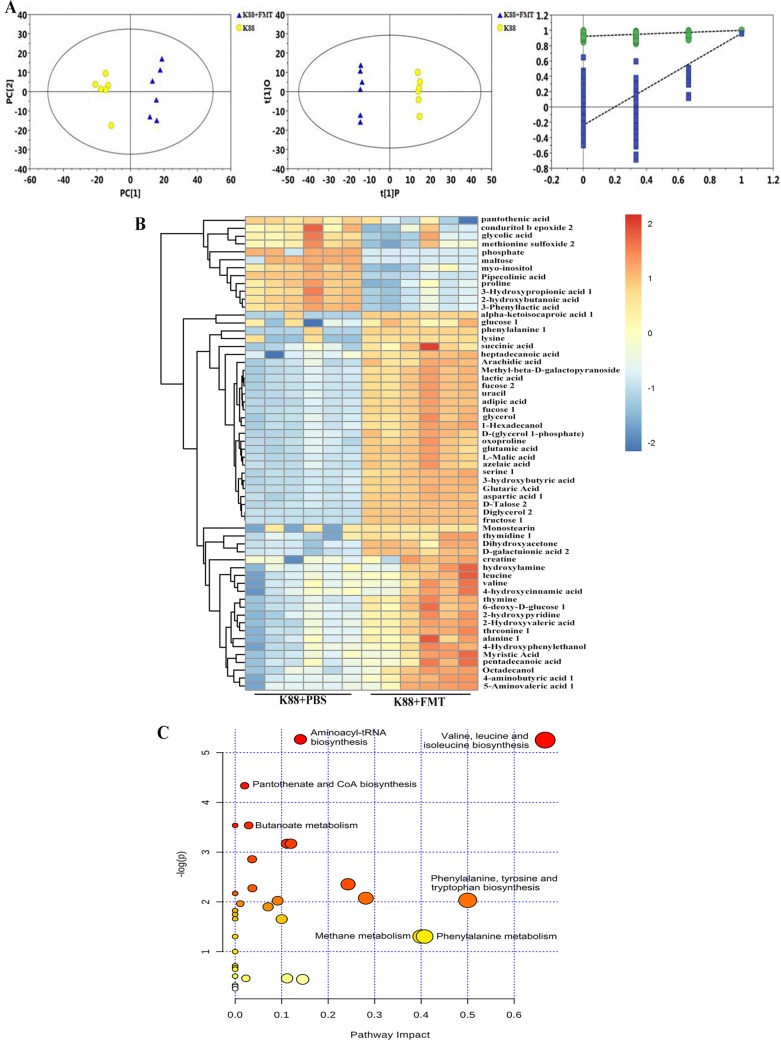
Statistical comparison of metabolites and analysis of differential metabolites and key metabolic pathways. (A) PCA score plot, OPLS-DA score plot, and permutation test plot of PLS-DA derived from the GC-TOF/MS metabolite profiles. Blue represents the infected piglets treated with exogenous fecal microbiota, and yellow represents the infected piglets without exogenous fecal microbiota intervention. The green circle represents the *R*^2^ value, the blue square represents the *Q*^2^ value, the green line represents the regression line of *R*^2^, and the blue line represents the regression line of *Q*^2^. (B) Heat map of hierarchical clustering analysis. The light blue boxes indicate an expression ratio less than the mean, and the dark red boxes denote an expression ratio greater than the mean. Tree clusters and their shorter Euclidean distances indicate higher similarities. (C) Metabolic pathway analysis of biomarker metabolites. The *x* axis represents the pathway impact, and the *y* axis represents the pathway enrichment. Larger sizes and darker colors represent higher pathway enrichment levels and higher pathway impact values, respectively.

To determine the differential levels of metabolites in intestinal lumen of E. coli K88-infected piglets following FMT, we conducted metabolomic analysis by gas chromatography-time of flight mass spectrometry (GC-TOF/MS). The typical total ion chromatograms [TICs] of the K88-plus-PBS group and the K88-plus-FMT group are shown in Fig. S4. The significant separation of clusters between the groups of infected piglets treated with or without exogenous fecal microbiota was evidenced by principal-coordinate analysis (PCA) score plot, orthogonal projections to latent structures-discriminate analysis (OPLS-DA) score plot, and permutation test plot of PLS-DA derived from the GC-TOF/MS metabolite profiles of colonic lumen (Fig. 7A). The *R*^2^*X* value of the PCA model representing the explained variance was 0.513, and the *Q*^2^ value representing the predictability of the model was 0.961. OPLS-DA also showed clear separation and discrimination between the two groups, evidenced by *R*^2^*Y* = 1 and *Q*^2^ = 0.961. Meanwhile, the permutation test (*R*^2^ = 0.921, *Q*^2^ = −0.239) assessed the robustness of the model.

10.1128/mSystems.00137-18.4 FIG S4 Typical total ion chromatograms (TICs) obtained from GC-TOF/MS analysis. (A) TICs of K88-plus-PBS group. (B) TICs of K88-plus-FMT group. Download FIG S4, TIF file, 2.8 MB. Copyright © 2018 Cheng et al. 2018 Cheng et al. This content is distributed under the terms of the Creative Commons Attribution 4.0 International license.

Metabolomics results showed that there were 58 metabolites in the colonic lumen, mainly belonging to amino acids, carbohydrates, lipids, organic acids, etc., that differed between the K88-plus-PBS group and the K88-plus-FMT group (Fig. 7B). The significantly differential metabolites of two groups are shown in Table S3. Lactic acid, succinic acid, valine, and leucine levels were enriched whereas maltose, pantothenic acid, and inositol levels were reduced in the recipient piglets compared with the infected piglets without exogenous fecal microbiota intervention. Among these, a total of 12 kinds of amino acids, including valine, leucine, phenylalanine, lysine, serine, alanine, and aspartic acid, were identified, and the levels of valine, leucine, serine, aminobutyric acid, phenylalanine, aminovaleric acid, alanine, aspartic acid, lysine, and creatine were increased. Seven differential metabolic pathways associated with amino acid metabolism, methane metabolism, aminoacyl-tRNA biosynthesis, pantothenate, and coenzyme A (CoA) biosynthesis were enriched (Fig. 7C). These differential amino acids were mainly enriched with respect to two metabolic pathways, namely, valine, leucine, and isoleucine metabolism and phenylalanine, tyrosine, and tryptophan metabolism.

10.1128/mSystems.00137-18.8 TABLE S3 Significantly differential metabolites in the colonic lumen. Download Table S3, DOCX file, 0.0 MB. Copyright © 2018 Cheng et al. 2018 Cheng et al. This content is distributed under the terms of the Creative Commons Attribution 4.0 International license.

### Intestinal morphology and barrier.

**FIG 8:**
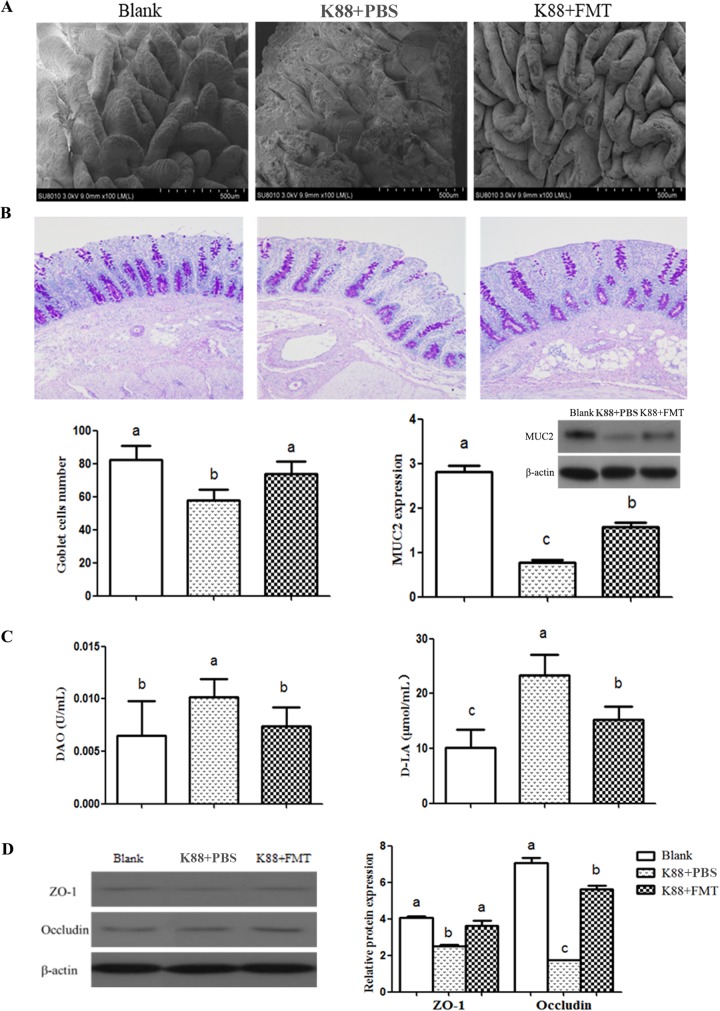
Intestinal morphology and barrier analysis of piglets. (A) The morphology of jejunum villi observed by scanning electron microscope. (B) The PAS staining of goblet cells (100×), the number of goblet cells and relative protein expression of MUC2 in colonic mucosa. (C) DAO activity and d-LA content in serum. (D) Relative levels of protein expression of ZO-1 and occludin in the colonic mucosa. Data are expressed as means ± standard deviations (SD). *, *P < *0.05 (*n* = 3). The letters a, b, and c represent the level of statistical significance of the difference between the groups. Identical letters indicate that the difference is not significant; different letters indicate that the difference is significant.

To determine the protective effect of FMT on the intestinal epithelium of infected piglets, the intestinal villi were observed through a scanning electron microscope (SEM) and the levels of goblet cells and MUC2 protein that they secreted were measured using periodic acid-Schiff (PAS) staining and Western blotting, respectively. The integrity of mechanical barrier was determined by analysis of serum diamine oxidase (DAO) activity and d-lactate (d-LA) content and intestinal TJ expression. The jejunal villi of infected piglets in the K88-plus-PBS group had severe damage, characterized by villous atrophy, inflammation, and blunting. However, there was no significant difference between the blank group and the K88-plus-FMT group with respect to the levels of damage (Fig. 8A), demonstrating that such damage of jejunal villi caused by E. coli K88 infection can be effectively rescued by FMT. The PAS staining of goblet cells and the protein expression of MUC2 in colonic mucosa are shown in Fig. 8B. Compared with the infected piglets, the number of goblet cells in colonic mucosa of recipient piglets increased significantly (*P < *0.05). Similarly, the protein expression of MUC2 also increased (*P < *0.05). These results indicate that FMT enhanced the mucosal barrier and protected the intestinal epithelium. The serum DAO activity and d-lactate content of recipient piglets were reduced significantly (*P < *0.05) (Fig. 8C), indicating that the intestinal permeability was decreased after FMT treatment. Data representing the protein expression of TJs in the colonic mucosa are shown in Fig. 8D. The protein expression of ZO-1 and occludin in the colonic mucosa of recipients were higher than in the colonic mucosa of infected piglets (*P < *0.05), suggesting that the exogenous fecal microbiota intervention enhanced the levels of tight junction proteins of the intestinal epithelium.

## DISCUSSION

The effectiveness of FMT in treatment of CDI and inflammatory bowel disease (IBD) has bolstered interest in study of its potential application. The alteration of gut microbial structure might play a key role in the process of FMT functioning. Our previous study (17) showed that introducing exogenous fecal microbiota changes the composition of the existing intestinal microbiota. In the present study, we observed that several specific pathways, including those associated with autophagy, protein processing, lipid metabolism, oxidative stress, and inflammation, were altered in the intestinal mucosa following FMT. Autophagy within the intestinal epithelium could maintain intestinal barrier integrity and limit intestinal inflammation by preventing the passage of invasive bacteria through the epithelium (18). Defects in autophagy may cause severe intestinal immunopathological damage (19). The induction of autophagy involves many proteins and multiple signaling pathways. Autophagy-related (Atg) proteins have central functions in the initiation and formation of autophagosomes (20). Mammalian Atg8 homologs consist of LC3 proteins and GABARAPs, all of which are known to be involved in canonical autophagy. Among these, LC3s have an important role in recruitment of cytosolic receptors, while GABARAPs in general promote transport and recruitment of membrane-bound factors required for autophagosomal maturation (21). When autophagy is activated, LC3B is cleaved to proteolytically derived LC3-II. During autophagy, other Atg proteins, including Atg7, Atg5, and Atg3, are required for autophagosome formation. Moreover, several signaling pathways, including the AKT (alpha serine/threonine kinase)/FoxO (forkhead box O), AMPK (AMP-activated protein kinase), and mTOR (mammalian target of rapamycin) pathways, modulate autophagy at different autophagosome formative stages (20). In this study, these autophagy-related proteins and signaling pathways in colonic mucosa were changed in piglets following FMT.

FoxOs are essential regulators of cellular homeostasis, as they play roles in homeostatic pathways, including regulation of autophagy, glucose and lipid metabolism, and oxidative defense activity (22). Transcriptional activity of FoxOs is inhibited by insulin and insulin-like growth factor signaling through direct phosphorylation mediated by AKT. In a recent study, a liver-specific triple knockout of FoxO1, FoxO3, and FoxO4 revealed a role for FoxOs in both autophagy and lipid metabolism (23). In addition, FoxO1-mediated autophagy was required for NK cell development, a major component of the innate immune system (24). In this study, the protein expression levels of FoxO1a and FoxO3a and Atg proteins such as GABARAP, LC3II/LC3I, and Atg7 were seen to increase in the colonic mucosa of recipient piglets. The FoxO signaling pathway was enriched per the KEGG pathway analysis results, indicating that exogenous fecal microbiota intervention increased FoxO-mediated autophagy in the intestinal mucosa of the recipient.

AMPK, a crucial cellular energy sensor, directly senses low energy status and is activated when cellular AMP or ADP levels increase. It is also activated by increases in the levels of LKB1 (also known as STK11), the major upstream AMPK threonine 172 kinase, through a noncanonical pathway triggered by reactive oxygen species (ROS) and DNA damage (25). In this study, the results showed that LKB1 and AMPK in the colonic mucosa were at low levels, which indicates that the intestine was in a state with sufficient cellular energy, no oxidative stress, and no DNA damage. mTOR is a highly conserved serine/threonine protein kinase that exists in two distinct complexes, mTORC1 and mTORC2, and mTORC1 has a negative regulatory role in autophagy. TSC2 inhibits Rheb, the upstream activator of mTORC1, while AKT and AMPK can activate or inhibit mTORC1 function by inhibiting or activating TSC2, respectively (20, 26). Our study showed increased phosphorylation of mTORC1 and inhibition of AKT, TSC2, and AMPK in the colonic mucosa, following FMT. Taking the results together, the activation of mTORC1 and the inhibition of AMPK indicated that the intestine was in a state of nutritional and energetic sufficiency. Mucosal autophagy was performed by regulating the expression of autophagy-related proteins such as GABARAP, LC3B, and Atg 7 in the FoxO signaling pathway rather than in starvation-induced and mTOR-dependent manners.

The maintenance of the intestinal epithelial redox environment is crucial to the activities of key physiological processes, including digestion and absorption, cell proliferation and apoptosis, and immune responses (27). MnSOD, also known as SOD2, is a vital antioxidant protein involved in oxidative stress. In this study, SOD2 expression was increased in the colonic mucosa following FMT. To counteract the adverse consequences of oxidative stress, cells have several oxidative defense mechanisms, including those controlled by the FoxO transcription factors. FoxOs resists physiologic oxidative stress responses by regulating the transcription of antioxidant enzymes such as SOD2 and catalase (28). Dysfunction of autophagy results in increased oxidative stress (29). In addition, antioxidants could exert their protective role by increasing the autophagy level. *tert*-Butylhydroquinone (tBHQ), a well-known antioxidant, could protect hepatocytes against lipotoxicity via inducing autophagy (30). In this study, simultaneous increases in the levels of autophagy and antioxidant enzymes were found, suggesting that exogenous fecal microbiota intervention increased FoxO-mediated autophagy to elevate the resistance to oxidative stimuli.

NF-κB is one of the most important transcription factors, and its activation is essential in signaling induced by pathogen- or damage-associated molecular patterns and cellular stresses. Once NF-κB is activated by pathogenic stimuli, comprehensive responses are induced, including overproduction of cytokines, such as tumor necrosis factor alpha (TNF-α), IL-1β, and IL-6 (31). Phosphorylation of transcription factor IFN regulatory factor 3 (IRF3) leads to the initiation of type I IFN production (32). In this study, the results showed that the level of IRF3 expression was depressed and that the protein expression levels of cytokines IFN-γ, IL-1β, and NF-κB were also depressed in the colonic mucosa following FMT. Modulation of NF-κB activation could be an important approach to reduce cellular injury. In addition, the process of autophagy has displayed a regulatory role in the mucosal immune system (33). These results indicated that exogenous fecal microbiota may inhibit NF-κB activation in intestinal mucosa by increasing mucosal autophagy.

Piglets are susceptible to various pathogens because of their unstable gut microbiota and immature immune system. Enterotoxigenic Escherichia coli is one of the major infectious factors causing diarrhea in piglets (34). In experiment II, an increase in diarrhea symptoms and body temperature was observed after E. coli K88 infection. When infected piglets received exogenous fecal microbiota, the diarrhea incidence decreased and body temperature gradually returned to normal. Similar results have been observed in diarrhea patients with C. difficile after they received a transplant of a fecal microbiota suspension from healthy humans (35). Our results confirmed that the diarrhea caused by E. coli K88 infection was effectively relieved by FMT.

Studies in C. difficile patients showed that the microbiota diversity was enhanced significantly via FMT (36). Similarly, we observed that the diversity of intestinal microbiota increased in E. coli K88-infected piglets following FMT. *Proteobacteria* is a major phylum of Gram-negative bacteria, which includes a wide variety of pathogens, such as *Escherichia*, *Salmonella*, *Vibrio*, and *Helicobacter*. In the state of intestinal microbiota dysbiosis, colonic epithelial oxidation increases and the anaerobic environment is affected; thus, facultative anaerobic *Proteobacteria* amplification is favored (37). A study of mice administered enterotoxigenic E. coli (ETEC) bacteria showed an increase in the abundance of *Enterobacteriaceae* (38). Our study also found a significant increase of *Enterobacteriaceae* abundance in the infected piglets. *Lactobacillus*, *Phascolarctobacterium*, and *Parabacteroides* can produce short-chain fatty acids (SCFAs). *Vibrio succinicus* can assist the host in metabolizing long-chain fatty acids and in producing organic acids such as acetic acid and succinic acid, which could play an inhibitory role in the growth of intestinal pathogenic bacteria (39). In the present study, the abundances of beneficial bacteria such as *Lactobacillus*, *Phascolarctobacterium*, *Parabacteroides*, and *Vibrio succinicus* in the intestine were increased after infected piglets received exogenous fecal microbiota, while those of harmful bacteria such as *Proteobacteria* and *Enterobacteriaceae* were reduced. These results indicate that the intestinal microbiota was diversified in the infected piglets following FMT through the inhibition of proliferation of harmful bacteria and the increase in the levels of beneficial bacteria.

To investigate the change in microbiota metabolic function caused by FMT, functional metagenomics prediction analysis was performed. In this study, the abundances of genes related to linoleic acid metabolism, fructose and mannose metabolism, lysine biosynthesis, and bisphenol degradation were enhanced and the abundance of genes related to biotin (vitamin H) metabolism was depressed in the E. coli K88-infected piglets following FMT. Linoleic acid plays a critical physiological role as an essential fatty acid. Various fatty acids such as long-chain fatty acids (e.g., linoleic, linolenic, and oleic acids) and medium-chain fatty acids (capric and lauric acids) are endogenous ligands of GPR40 (free fatty acid receptor 1 [FFA1]), with linoleic acid exhibiting the highest affinity for GPR40. 10-Hydroxy-cis-12-octadecenoic acid, a metabolite of linoleic acid, could alter the expression of TJ-related molecules such as occludin, atactin-1, and myosin light-chain kinase and improve intestinal barrier function by the GPR40-MEK-ERK pathway (40). Our results indicated that enhanced linoleic acid metabolism contributed to the improvement of gut barrier integrity.

The host and its gut microbiota are linked by the metabolites secreted by the microbiota. In the present study, the detected differential amino acids were enriched in valine, leucine, and isoleucine metabolism as well as in phenylalanine, tyrosine, and tryptophan metabolism. Additionally, valine and leucine were the differentially abundant metabolites. Branched-chain amino acids (leucine, valine, and isoleucine) can promote the expression of antimicrobial peptides and immunoglobulins in the intestine and can improve the intestinal immune barrier function (41). In this study, the levels of lactic acid and succinic acid in the intestinal lumen were increased after infected piglets received exogenous fecal microbiota. Lactic acid is the main product produced by *Lactobacillus* utilizing carbohydrates, and it inhibits the reproduction of pathogenic bacteria such as pathogenic Escherichia coli. Succinic acid is produced by bacteria such as Bacteroides fragilis, *Prevotella*, and *Vibrio succinicus*, and it acts as a glucose precursor to activate the gluconeogenesis pathway in the intestine to maintain normal blood glucose levels (42). Therefore, the increase in the levels of lactic acid and succinic acid was consistent with the increase of *Lactobacillus* and *Vibrio succinicus* abundance in the intestinal lumen, which might be beneficial with respect to relieving intestinal barrier injury in infected piglets following FMT.

The integrity of the gut barrier is crucial for maintaining the normal function of the epithelium and preventing the invasion of pathogenic bacteria. The present results showed that while intestinal villi of E. coli K88-infected piglets were extensively damaged, the damaged villi were effectively repaired following FMT. Goblet cells, mucus, and mucin in the intestinal epithelium play key immune regulatory roles in the intestine (7). Studies showed the possible role that *Lactobacillus* plays in maintenance of intestinal integrity by upregulating mucosal MUC2 expression levels, and butyrate could also promote MUC2 expression (43). In this study, increased levels of *Lactobacillus* abundance, butyrate metabolism, goblet cells, and MUC2 expression were observed in the E. coli K88-infected piglets following FMT. DAO and d-lactic acid are considered sensitive markers for monitoring the intestinal barrier permeability (44). We found that both serum DAO activity and d-lactic acid content were reduced in the infected piglets following FMT, which was consistent with the aforementioned conclusion that the increase in *Lactobacillus* levels improved intestinal permeability. TJs, the main transmembrane proteins in the intestinal epithelial cells, are directly responsible for intestinal barrier (45). ZO-1 and occludin expression levels were enhanced in the colonic mucosa in the infected piglets following FMT. These results indicated that exogenous fecal microbiota intervention relieved intestinal barrier injury and enhanced the gut barrier integrity of infected piglets.

**FIG 9:**
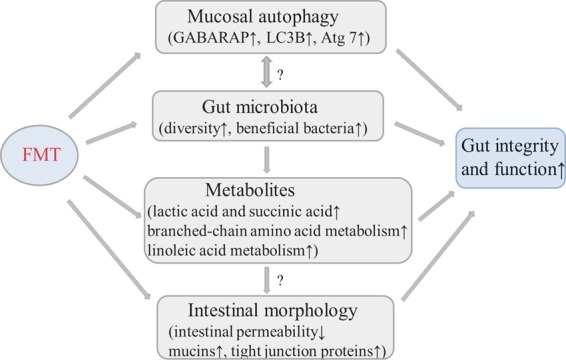
Integrative diagram showing the main results obtained in the current work. The up arrows (↑) indicate increasing effects, and the down arrow (↓) indicates decreasing effects. The question marks (?) indicate possible relationships to be further explored in future studies.

Fig. 9 summarizes and integrates the main results obtained in the current work. The results seen with mucosal proteomes showed that the levels of autophagy-related proteins in the recipient were increased whereas the levels of the proteins related to inflammation response were decreased. In addition, epithelial injury was alleviated in the E. coli K88-infected piglets following FMT. Intestinal morphology was improved, and the intestinal villi were smooth and integrated relatively well, which coincided with the depression of intestinal permeability and the enhancement of mucins and mucosal TJ expression in the recipient. 16S rRNA gene sequencing and metabolomic analysis revealed that the abundances of microbiota and metabolites beneficial to gut were upregulated. Taken together, the results showed that FMT triggered intestinal mucosal protective autophagy and alleviated epithelial injury through alteration of the gut microbial composition.

In conclusion, studies have shown therapeutic benefits of FMT in multiple diseases in human and animal models. Given these profound effects on health and disease, there is strong interest in discovering the interplay between host and microbiota to regulate the gut microbiota for therapeutic purposes. Here, we demonstrated that FMT triggered mucosal protective autophagy during the colonization process and alleviated the gut barrier injury caused by E. coli K88 through reconstitution of microbiota. Therefore, our study showed that a beneficial host-microbiota interrelation might be established following FMT, which might provide insight into the potential of FMT to be an effective therapeutic strategy for various intestinal diseases in humans and animals.

## MATERIALS AND METHODS

### Preparation of fecal microbiota suspension and bacterial strains.

Jinhua pigs, representing a local breed in Zhejiang province, China, that had had no antibiotics or medicinal feed additives treatment within 3 months were used in this study as fecal donors. The fecal suspension was prepared as previously described (17, 46). Enterotoxigenic E. coli K88 was purchased from the Institute of Veterinary Drugs Control (Beijing, China).

### Animals and treatments.

This experiment was approved by the Animal Care and Use Committee of Zhejiang University (permit number SYXK 2012-0178), and all experimental procedures were performed in accordance with the institutional guidelines for animal research.

In experiment I, a total of 6 litters (9 to 11 piglets per litter) of DLY (Duroc × Landrace × Yorkshire) newborn piglets with the same birth day and parity were selected. The piglets were randomly divided into two groups with 3 litters in each group, namely, an FMT group and a control group. The two groups were inoculated orally with a fecal microbiota suspension and phosphate-buffered saline (PBS), respectively. The specific process was performed as previously described (17). The trial lasted for 14 days.

In experiment II, a total of 18 DLY piglets (average body weight, 9.67 ± 0.58 kg) were randomly assigned to two groups, namely, a blank group (*n* = 6) and a K88 group (*n* = 12). All piglets were fed antibiotic-free feed during the 21-day trial. The piglets in the K88 group were inoculated with 100 ml of E. coli K88 bacterial suspension at h 1300 to h 1400 from day 15 to day 17. The piglets in the K88 group were then inoculated orally with 100 ml of PBS or fecal microbiota suspension from day 18 to day 20, forming the K88-plus-PBS group (*n* = 6) or K88-plus-FMT group (*n* = 6), respectively. The piglets in the blank group were not administered any treatment. The diet was designed to meet requirements recommended by the National Research Council (NRC) (47). All piglets had free access to feed and water. The mental state, diarrhea incidence, and rectal temperature of piglets were recorded after E. coli K88 infection. The body weight of each piglet was measured at the beginning and the end of the experiment. (The experiment design is shown in Fig. S5 in the supplemental material.)

10.1128/mSystems.00137-18.5 FIG S5 Experimental design used in this study. (A) Experimental design for experiment I. Six litters of DLY newborn piglets were randomly divided into two groups, namely, an FMT group and a control group, with 3 litters in each group and inoculated orally with 1.5 ml of fecal microbiota suspension and PBS, respectively. (B) Experimental design for experiment II. A total of 18 DLY piglets were randomly assigned to two groups, namely, a blank group and a K88 group. The piglets in the K88 group were inoculated with 100 ml of E. coli K88 bacterial suspension from day 15 to day 17. The piglets in two K88 groups, namely, a K88-plus-PBS group and a K88-plus-FMT group, were then inoculated orally with 100 ml of PBS and a fecal microbiota suspension, respectively, from day 18 to day 20. The piglets in the blank group were not administered any treatment. Download FIG S5, TIF file, 0.1 MB. Copyright © 2018 Cheng et al. 2018 Cheng et al. This content is distributed under the terms of the Creative Commons Attribution 4.0 International license.

### Sample collection.

In experiment I, at the end of the experiment (day 15), 6 piglets were randomly selected from the FMT group and the control group (total, 12 piglets) to be slaughtered. The mucosa samples of colon were scraped carefully on an ice bag using a glass microscope slide. Then, the samples were transferred into Eppendorf tubes, snap-frozen in liquid nitrogen, and stored at −80°C.

In experiment II, at day 22, all piglets were slaughtered. The serum and colonic contents of samples were collected, snap-frozen in liquid nitrogen, and stored at −80°C for further analysis. The samples of jejunum and colon segments were collected and fixed in 10% buffered formalin at 4°C for observation by light microscopy. The jejunum samples for electron microscopy assessment were fixed in a 2.5% glutaraldehyde solution at 4°C. The colon samples for periodic acid-Schiff (PAS) staining were fixed in 4% paraformaldehyde for 24 h at room temperature and then processed for paraffin embedding.

### Determination of intestinal mucosal proteome profile. (i) Protein extraction, digestion, and iTRAQ labeling.

In experiment I, an isobaric tag was applied for relative and absolute quantification (iTRAQ)-based quantitative proteomic analysis. The colonic mucosal samples were used for protein extraction according to a method reported previously (48). The supernatants were stored at −80°C for iTRAQ and Western blot analysis. The protein suspensions were digested with trypsin (Promega, Madison, WI) overnight at 37°C. The peptides were dried by vacuum centrifugation and reconstituted in 0.5 M TEAB (Applied Biosystems, Italy). The tryptic peptides were labeled with iTRAQ tags (for controls, tags 113, 114, and 115; for FMT, tags 116, 117, and 118) according to the instructions of an iTRAQ reagent-8plex kit (AB Sciex). All labeled samples were incubated for 2 h at room temperature and then mixed and dried by vacuum centrifugation.

### (ii) SCX fractionation and LC-MS/MS determination.

Strong-cationic-exchange chromatography (SCX) was performed according to the manufacturer’s instructions. The iTRAQ-labeled peptides mixtures were eluted, a total of 10 fractions were collected, and then each fraction was desalted on a C_18_ column (Empore SPE C_18_ cartridges; Sigma) and dried by vacuum centrifugation. Each SCX fraction was redissolved in buffer A (2% ACN, 0.1% FA) and then centrifuged at 20,000 × *g* for 10 min. The peptide mixture was loaded onto a reverse-phase trap column (Thermo Scientific Acclaim PepMap 100) connected to the C_18_ reversed-phase analytical column (Thermo Scientific Easy Column) in buffer A and eluted with a 50-min gradient at a flow rate of 300 nl/min from 0% to 35% buffer B (84% ACN, 0.1% FA), followed by a 5-min linear gradient to 100% buffer B. After liquid-phase separation, liquid chromatography-tandem mass spectrometry (LC-MS/MS) analysis was performed on a Q Exactive mass spectrometer (Thermo Scientific) that was coupled online to Easy nLC (Thermo Fisher Scientific). The mass spectrometer was operated in positive ion mode. MS data were acquired for the 10 most abundant precursor ions from the MS survey scan (300 to 1,800 *m*/*z*) for high-energy collision dissociation fragmentation. The specific process was performed following a method described by Zhu et al. (49).

### Sequence database searching and data analysis.

MS/MS data for iTRAQ protein identification and quantitation were analyzed using Proteome Discoverer 1.4 (Thermo Fisher Scientific, Germany) and searched using the Mascot engine (version 2.2; Matrix Science, United Kingdom) against the Uniprot Sus_scrofa database with the following parameters: type of search, MS/MS ion search; maximum number of missed cleavages, 2; fixed modifications, carbamidomethyl (C), iTRAQ 8plex (N-term), and iTRAQ 8plex (K); variable modifications, oxidation (M) and iTRAQ 8plex (Y); peptide mass tolerance, ± 20 ppm; fragment mass tolerance, 0.1 Da. Identified peptides had an ion score above the threshold of peptide identity established by Mascot, and peptide identifications in which at least one such unique peptide match was specific for the protein were accepted with a FDR value of ≤1%. A quantitative protein with a ratio value of >1.2 or <0.83 and a *P* value of ≤0.05 was considered to be a differentially expressed protein. The sequence data of differentially expressed proteins were retrieved in batches from the UniProtKB database (Release 2016_10) in FASTA format. Differentially expressed proteins were further analyzed by using Blast2GO (version 3.3.5) for GO mapping and annotation. The studied proteins were then subjected to a BLAST search against the online Kyoto Encyclopedia of Genes and Genomes (KEGG) database to retrieve their knockouts (KOs) and were subsequently mapped to pathways in KEGG.

### Validation of differentially expressed proteins.

Mucosal proteins from individual samples were separated by SDS-PAGE and transferred to polyvinylidene difluoride (PVDF) membranes (Millipore, USA). The membranes were blocked in Tris-buffered saline with Tween 20 (TBST)–5% nonfat milk for 2 h at room temperature, and then the membranes were incubated with primary antibodies overnight at 4°C. The following antibodies were used for Western blot analysis: anti-AKT (Pan) (CST 4691) (1:1,000), anti-phospho-AKT (Ser473) (CST 4060) (1:1,000), anti-FoxO1a (Abcam ab70382) (1:4,000), anti-FoxO3a (Abcam ab12162) (1:2,500), anti-GABARAP (Abcam ab227732) (1:800), anti-LC3B (Abcam ab229327) (1:1,000), anti-Atg7 (Proteintech 10088-2-AP) (1:500), anti-SOD2 (Abcam ab13534) (1:2,000), anti-phospho-NF-κB P65 (Ser536) (CST 3033), anti-AMPK alpha 1 (Abcam ab3759) (1:500), anti-phospho-AMPKa (Thr172) (CST2535) (1:1,000), anti-mTOR (Abcam ab2833) (1:1,000), anti-phospho-mTOR (Abcam ab84400) (1:500), anti-IFN-γ (Thermo Fisher MP701) (1:500), anti-IL-1β (Bio-Rad MCA23612) (1:200), and anti-glyceraldehyde-3-phosphate dehydrogenase (anti-GAPDH) (Abcam ab181602) (1:10,000). The membranes were then washed three times with TBST and subsequently incubated with the secondary antibodies (goat anti-mouse IgG [Thermo Pierce 31160] [1:5,000]) for 2 h at room temperature. The signals were detected by the use of a SuperSignal West Dura extended-duration substrate kit, and the protein bands were visualized using Image J analysis software (NIH).

### Analysis of intestinal microbiota structure and function. (i) DNA extraction and 16S rRNA amplicon sequencing.

In experiment II, DNA was extracted from approximately 100-mg samples of colonic lumen using a TIANamp stool DNA kit according to the manufacturer’s instructions. DNA was amplified by using the 341F-805R primer set (341F [5′-CCT ACG GGN GGC WGC AG-3′] and 805R [5′-GAC TAC HVG GGG TAT CTA ATC C-3′]), which targeted the V3-plus-V4 region of the bacterial 16S rRNA gene. PCR products were purified by using a GeneJET gel extraction kit (Thermo Scientific). Sequencing libraries were generated using a NEB Next Ultra DNA Library Prep kit for Illumina (NEB, USA) following the manufacturer’s recommendations, and index codes were added. The library quality was assessed using a Qubit@ 2.0 Fluorometer (Life Technologies, CA, USA) and an Agilent Bioanalyzer 2100 system. Lastly, the library was sequenced on an Illumina MiSeq platform and 250-bp paired-end reads were generated.

### (ii) Sequencing data analysis and functional metagenomics prediction.

QIIME software (version 1.8.0.) was used for sequence analysis, including the extraction of operational taxonomic units (OTUs), alpha diversity analysis, clustering analysis, linear discriminant analysis coupled with effect size (LEfSe) determinations, etc. OTUs were clustered using the average neighbor algorithm with a cutoff value of 97% similarity. Alpha diversity analysis included Shannon, Chao1, Observed species, and Simpson. LEfSe analysis was performed to identify the bacterial species with significant differences in abundance between groups (i.e., biomarkers). PICRUSt (phylogenetic investigation of communities by reconstruction of unobserved states) was used to predict the functional profiles of microbial communities. Two-sided Welch’s *t* tests and Benjamini-Hochberg FDR corrections were used in two-group functional metagenomics prediction analysis.

### Metabolomic analysis by GC-TOF/MS. (i) GC-TOF/MS determination.

The colonic-content samples in the K88-plus-PBS and K88-plus-FMT groups were prepared for gas chromatography-time of flight mass spectrometry (GC-TOF/MS) analysis according to a method described by Sun et al. (50). The extracted metabolite samples were injected into an Agilent 7890 GC-TOF/MS system equipped with an Agilent DB-5MS capillary column (J & W Scientific, CA, USA) (30 m by 250 μm; 0.25 μm pore size). The front inlet purge flow rate was 3 ml/min, and the column flow rate was 1 ml/min. The column temperature was first held at 50°C for 1 min and then ramped at 10°C/min to 290°C for 15 min. The voltage level (energy) was −70 eV in electron impact mode. The mass spectrometry data were acquired in full-scan mode over a range of 50 to 500 *m*/*z* at a rate of 20 spectra per second after a solvent delay of 366 s.

### (ii) Data analysis.

Primitive peaks analysis, data baseline filtering, calibration of baseline, park alignment, deconvolution analysis, peak identification, and integration of peak areas were performed using Chroma TOF 4.3X software of the Leco Corporation and the LECO-Fiehn Rtx5 database. The peak identification was performed using the RI (retention time index) method with an RI tolerance value of 5,000. After the data were imported into the SIMCA 14.1 software package (Umetrics, Umea, Sweden), principal-component analysis (PCA), orthogonal projections to latent structures-discriminate analysis (OPLS-DA), and permutation tests were performed to visualize the differences between the groups. The parameters *R*^2^*Y* and *Q*^2^ were used to assess the robustness and the predictive ability of the model. Variable-importance projection (VIP) values exceeding 1.0 were selected as representative of changed metabolites, and then the Student’s *t* test was used to evaluate the remaining variables. In addition, databases, including KEGG (http://www.genome.jp/kegg/) and NIST (http://www.nist.gov/index.html), were employed to search for the metabolic pathways.

### Determination of intestinal morphologies and barriers.

The intestinal morphologies of the jejunum in the blank, K88-plus-PBS, and K88-plus-FMT groups were observed under a SEM. The goblet cells stained with PAS stain were analyzed with respect to morphology and distribution by light microscopy according to a method described by van Es et al. (51). The protein expression levels of MUC2, ZO-1, and occludin in the colon were measured by Western blotting. The anti-MUC2 (Santa Cruz SC-13312) (1:100), anti-ZO-1 (Santa Cruz SC-8146) (1:200), and anti-occludin (Santa Cruz SC-8144) (1:500) antibodies were used for Western blot analysis. Serum DAO activity and d-lactate content were measured using the corresponding kit according to the manufacturer’s instructions.

### Statistical analysis.

Data from comparisons between two groups were analyzed statistically using the independent sample *t* test (SPSS 23.0) to conduct variance analysis. Data from comparisons among three groups were analyzed by one-way analysis of variance (ANOVA) followed by Tukey’s multiple-comparison test (SPSS 23.0). *P* values of ≤0.05 were considered statistically significant.
