# Supplementary figures and images for: Fecal Microbiota Transplantation Beneficially Regulates Intestinal Mucosal Autophagy and Alleviates Gut Barrier Injury
Source: mSystems. 2018 Oct 9;3(5):e00137-18. doi: 10.1128/mSystems.00137-18 (PMC6178585; doi:10.1128/mSystems.00137-18)

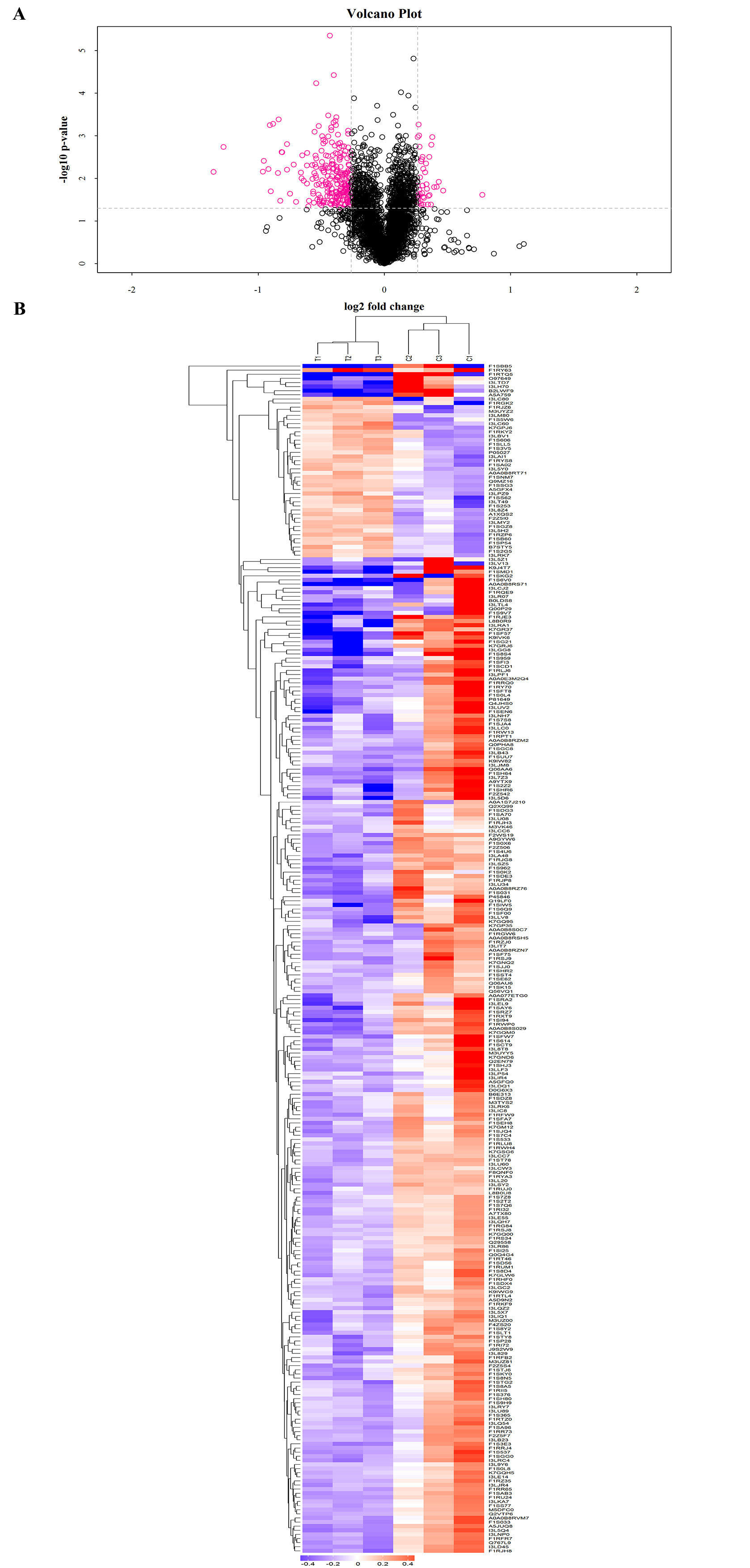

Supplement: FIG S1 [file sys005182272sf1.tif]

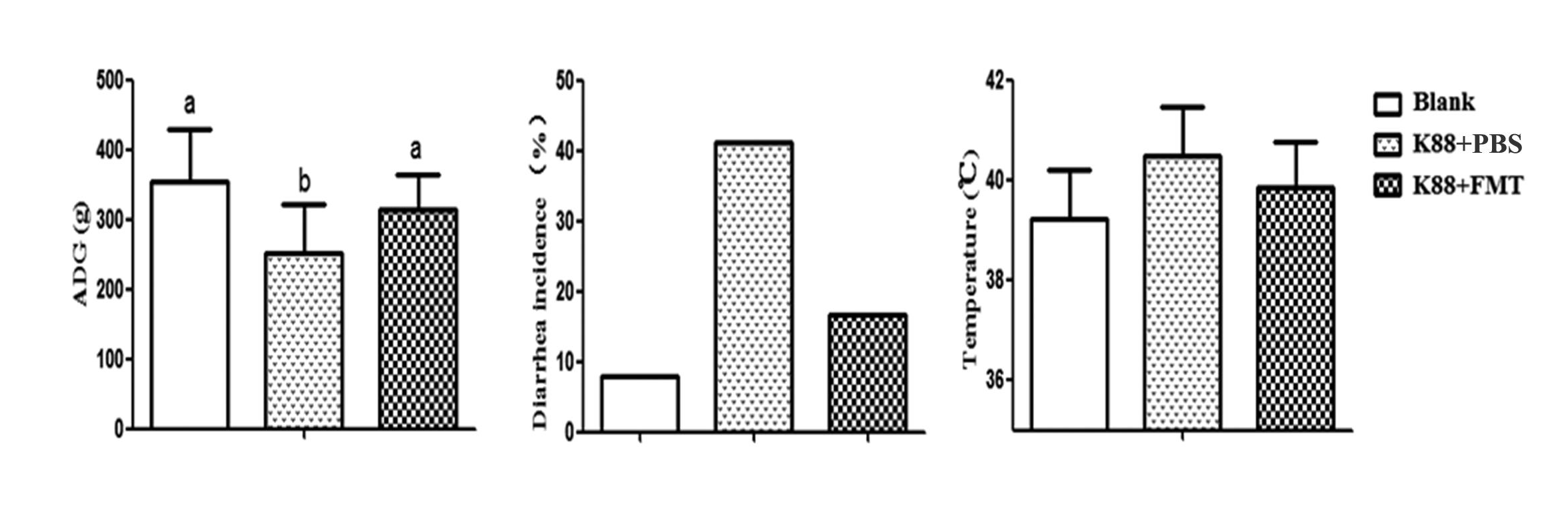

Supplement: FIG S2 [file sys005182272sf2.tif]

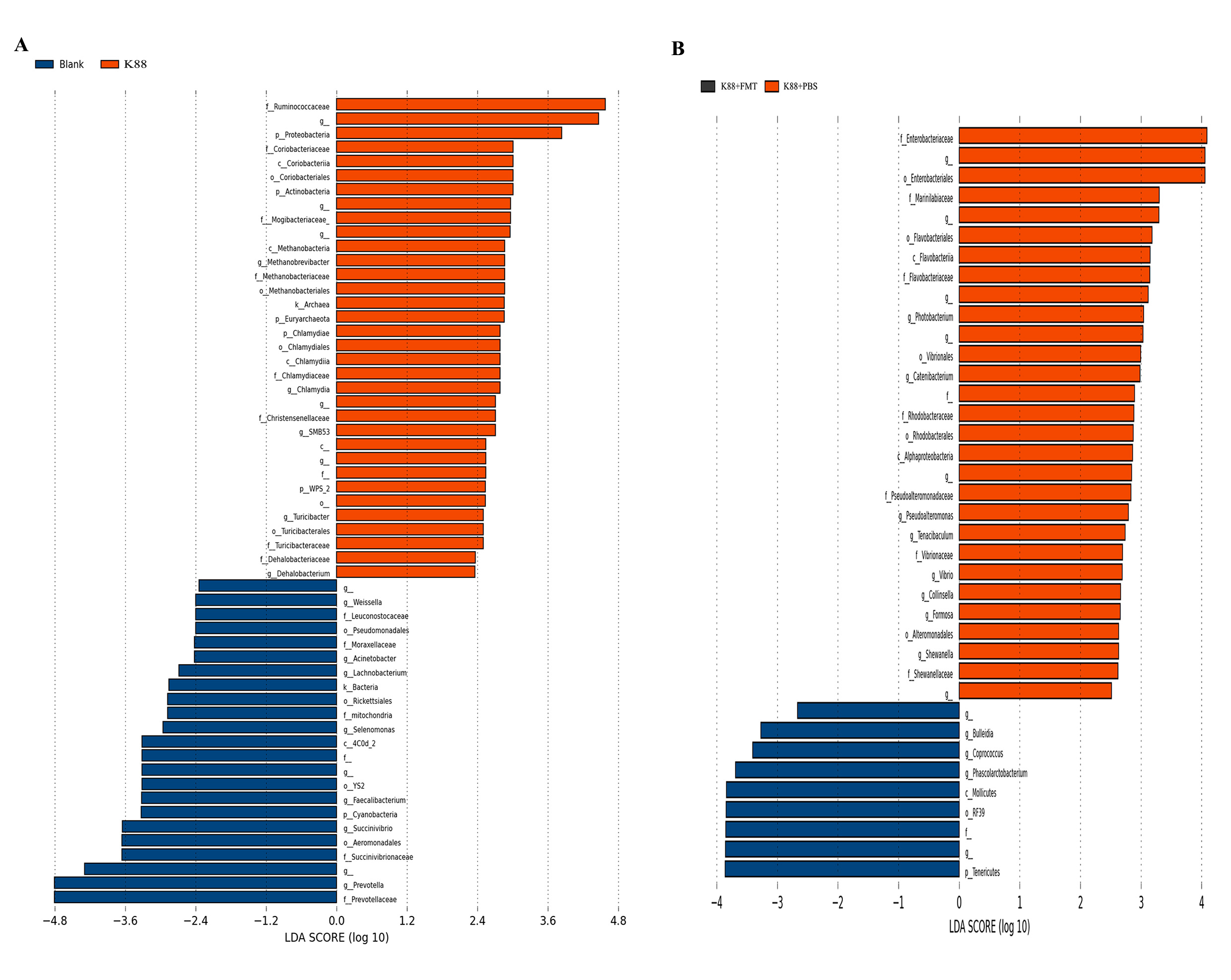

Supplement: FIG S3 [file sys005182272sf3.tif]

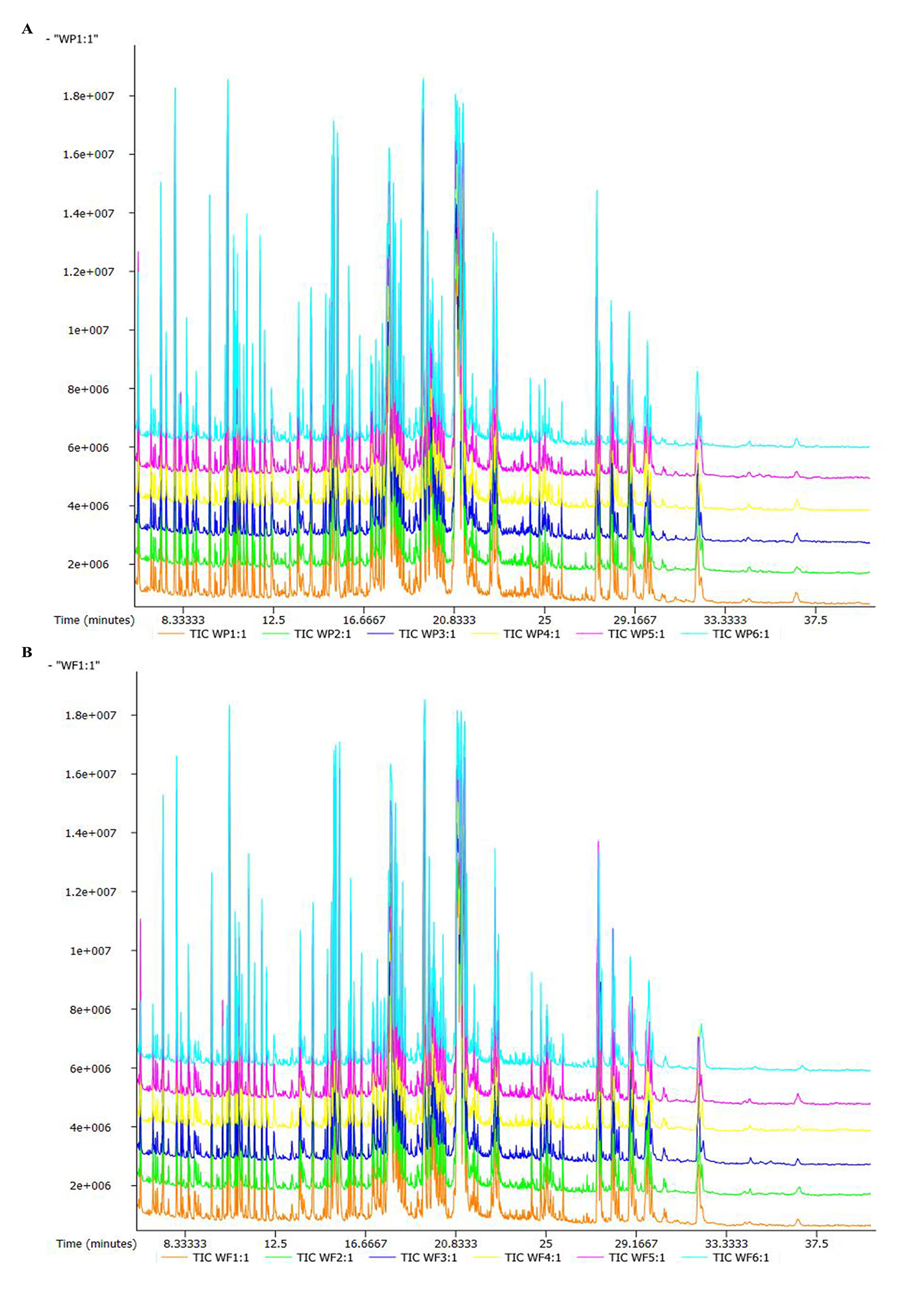

Supplement: FIG S4 [file sys005182272sf4.tif]

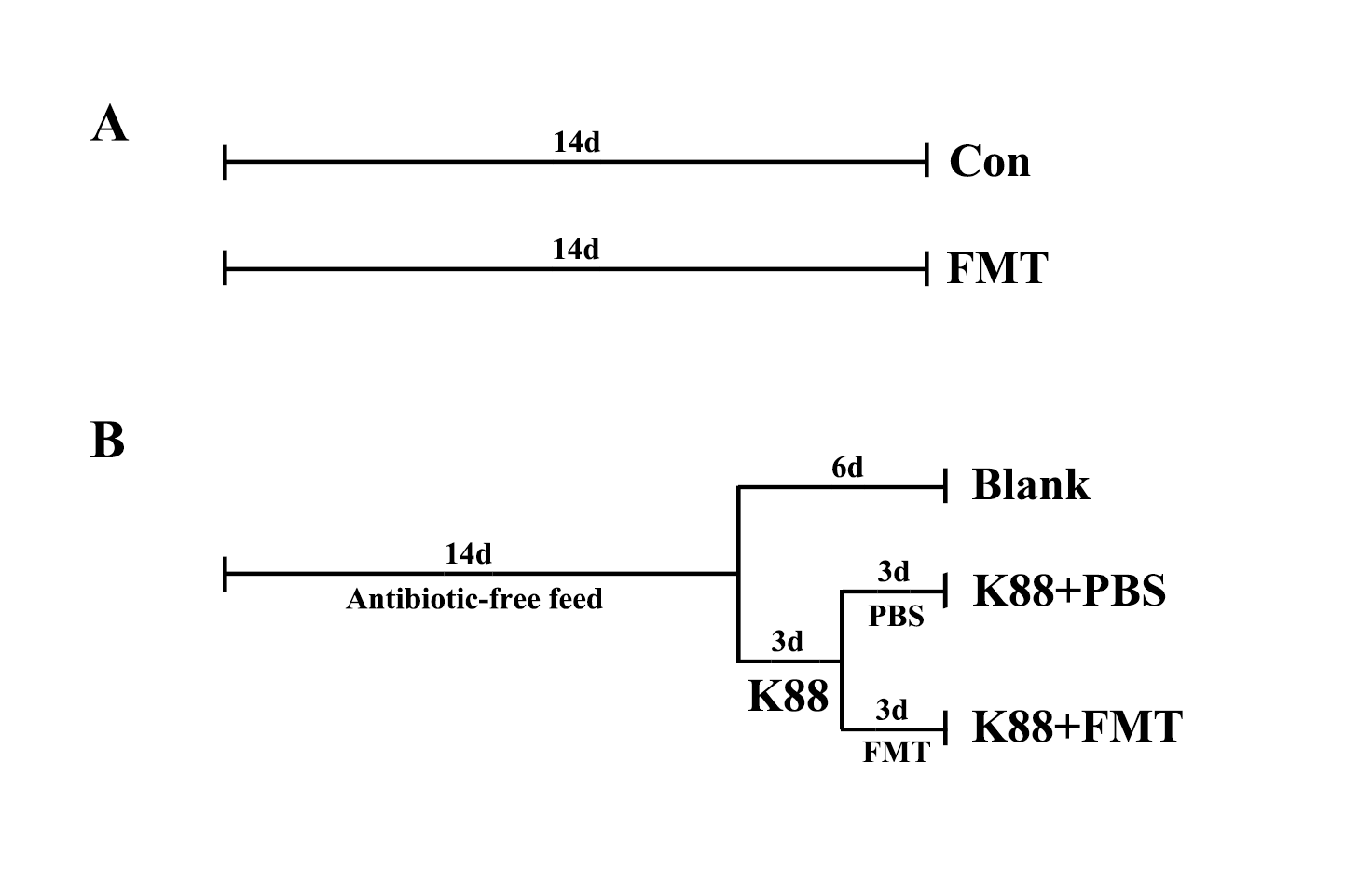

Supplement: FIG S5 [file sys005182272sf5.tif]
