# Supplementary material for: Fecal Microbiota Transplantation Beneficially Regulates Intestinal Mucosal Autophagy and Alleviates Gut Barrier Injury
Source: mSystems. 2018 Oct 9;3(5):e00137-18. doi: 10.1128/mSystems.00137-18 (PMC6178585; doi:10.1128/mSystems.00137-18)
Supplement: TABLE S2 [file sys005182272st2.docx]

**Supplementary Table 2 Pretreatment statistics and quality control of microbial sequencing data**

| Item | Blank | K88 | K88+PBS | K88+FMT |
| --- | --- | --- | --- | --- |
| Raw Reads | 84496 | 84988 | 83406 | 83710 |
| Clean Reads | 76828 | 77574 | 75507 | 76222 |
| Clean Q30 Bases Rate (%)^a^ | 86.94 | 86.81 | 86.88 | 86.98 |
| Total Aligned Tags | 20401 | 19605 | 20769 | 20054 |
| Aligned Rate (%)^b^ | 54.81 | 52.68 | 55.80 | 53.88 |
| Total Assembled Reads^c^ | 38399 | 38775 | 37739 | 38096 |
| Total Assembled Rate (%)^d^ | 99.96 | 99.97 | 99.96 | 99.96 |
| Average Assembled Length^e^ | 450.28 | 449.51 | 450.47 | 447.75 |
| Total OTUs Number | 971 | 982 | 938 | 1006 |

^a^ Clean Q30 Bases Rate (%), The ratio of bases with a quality of more than 30 (error rate less than 0.1%) in Clean Reads to the total base (Clean Reads).

^b^ Aligned Rate(%), The proportion of the matched sequence to the total stitched sequence.

^c^ Total Assembled Reads, The number of pairs of sequences that can be successfully stitched (due to sequencing errors, too long fragments, etc., so not every piece can be successfully stitched), also known as Tags.

^d^ Total Assembled Rate (%), The ratio of sequences that can be spliced into one piece to the total filtered sequence.

^e^ Average Assembled Length, When splicing into a complete segment, the average length of the two sequences after splicing.
