# Supplementary material for: Fecal Microbiota Transplantation Beneficially Regulates Intestinal Mucosal Autophagy and Alleviates Gut Barrier Injury
Source: mSystems. 2018 Oct 9;3(5):e00137-18. doi: 10.1128/mSystems.00137-18 (PMC6178585; doi:10.1128/mSystems.00137-18)
Supplement: TABLE S3 [file sys005182272st3.docx]

**Supplementary Table 3 Significant differential metabolites in the colonic lumen**

| **Metabolites and classification** | **Similarity^a^** | **K88+PBS** | **K88+FMT** | **VIP^b^** | **P-VALUE^c^** | **FC^d^** |
| --- | --- | --- | --- | --- | --- | --- |
| Aromatic Heteromonocyclic Compounds | | | | | | |
| thymine | 892 | 0.549 | 0.683 | 1.580 | <0.0001 | 1.244 |
| uracil | 887 | 0.807 | 1.102 | 1.645 | <0.0001 | 1.367 |
| 2-hydroxypyridine | 852 | 0.461 | 0.602 | 1.506 | 0.0001 | 1.307 |
| Amino Acids, Peptides, and Analogues | | | | | | |
| serine 1 | 856 | 0.338 | 0.555 | 1.653 | <0.0001 | 1.642 |
| 4-aminobutyric acid 1 | 831 | 0.063 | 0.124 | 1.458 | 0.0020 | 1.970 |
| phenylalanine 1 | 829 | 0.111 | 0.625 | 1.426 | 0.0055 | 5.644 |
| proline | 816 | 1.497 | 1.296 | 1.519 | 0.0000 | 0.866 |
| 5-Aminovaleric acid 1 | 813 | 0.381 | 0.512 | 1.367 | 0.0013 | 1.342 |
| alanine 1 | 758 | 6.328 | 6.769 | 1.384 | 0.0010 | 1.070 |
| valine | 751 | 2.605 | 2.767 | 1.202 | 0.0085 | 1.062 |
| aspartic acid 1 | 741 | 0.031 | 0.064 | 1.666 | <0.0001 | 2.063 |
| lysine | 732 | 0.210 | 0.700 | 1.196 | 0.0134 | 3.341 |
| Pipecolinic acid | 661 | 0.212 | 0.086 | 1.673 | 0.0000 | 0.404 |
| leucine | 703 | 2.367 | 2.564 | 1.355 | 0.0015 | 1.084 |
| creatine | 654 | 0.004 | 0.005 | 1.063 | 0.0208 | 1.148 |
| Organic Acids and Derivatives |  |  |  |  |  |  |
| 3-Hydroxypropionic acid 1 | 845 | 0.075 | 0.055 | 1.582 | <0.0001 | 0.737 |
| adipic acid | 650 | 0.029 | 0.047 | 1.654 | <0.0001 | 1.627 |
| 2-Hydroxyvaleric acid | 639 | 0.006 | 0.007 | 1.518 | 0.0001 | 1.225 |
| succinic acid | 781 | 0.835 | 1.083 | 1.415 | 0.0010 | 1.298 |
| 2-hydroxybutanoic acid | 776 | 0.079 | 0.061 | 1.608 | <0.0001 | 0.778 |
| Glutaric Acid | 715 | 0.045 | 0.088 | 1.660 | <0.0001 | 1.951 |
| azelaic acid | 713 | 0.018 | 0.030 | 1.648 | <0.0001 | 1.686 |
| glycolic acid | 625 | 0.233 | 0.169 | 1.134 | 0.0305 | 0.725 |
| L-Malic acid | 709 | 0.017 | 0.046 | 1.655 | <0.0001 | 2.644 |
| Carbohydrates and Carbohydrate Conjugates | | | | | | |
| glucose 1 | 673 | 0.003 | 0.006 | 1.064 | 0.0263 | 2.389 |
| fucose 2 | 827 | 0.534 | 0.891 | 1.646 | <0.0001 | 1.670 |
| fucose 1 | 826 | 1.716 | 2.810 | 1.660 | <0.0001 | 1.638 |
| glycerol | 805 | 1.363 | 1.722 | 1.615 | <0.0001 | 1.264 |
| maltose | 802 | 0.009 | 0.000 | 1.390 | 0.0044 | 0.000 |
| Dihydroxyacetone | 749 | 0.268 | 1.138 | 1.606 | 0.0005 | 4.250 |
| Aromatic Homomonocyclic Compounds | | | | | |  |
| 4-Hydroxyphenylethanol | 654 | 0.007 | 0.008 | 1.353 | 0.0014 | 1.151 |
| glutamic acid | 794 | 0.099 | 0.129 | 1.648 | <0.0001 | 1.308 |
| 4-hydroxycinnamic acid | 697 | 0.004 | 0.006 | 1.115 | 0.0162 | 1.407 |
| Lipids |  |  |  |  |  |  |
| Arachidic acid | 807 | 0.052 | 0.078 | 1.629 | <0.0001 | 1.512 |
| Myristic Acid | 797 | 0.395 | 0.456 | 1.243 | 0.0061 | 1.156 |
| 1-Hexadecanol | 746 | 0.133 | 0.183 | 1.625 | <0.0001 | 1.370 |
| Octadecanol | 684 | 0.073 | 0.097 | 1.458 | 0.0003 | 1.326 |
| alpha-ketoisocaproic acid 1 | 635 | 0.002 | 0.017 | 1.432 | 0.0015 | 6.909 |
| pentadecanoic acid | 619 | 1.072 | 1.156 | 1.265 | 0.0048 | 1.079 |
| Homogeneous Non-metal Compounds | | | | | | |
| phosphate | 724 | 0.077 | 0.000 | 1.409 | 0.0059 | 0.000 |
| hydroxylamine | 805 | 0.002 | 0.004 | 1.105 | 0.0403 | 2.533 |
| Aliphatic Homomonocyclic Compounds | | | | | |  |
| myo-inositol | 839 | 0.353 | 0.303 | 1.482 | 0.0001 | 0.857 |
| Aliphatic Heteromonocyclic Compounds | | | | | |  |
| oxoproline | 787 | 1.949 | 2.804 | 1.647 | <0.0001 | 1.439 |
| Aliphatic Acyclic Compounds | | | | | |  |
| pantothenic acid | 816 | 0.020 | 0.019 | 1.217 | 0.0167 | 0.912 |
| Organic acids |  |  |  |  |  |  |
| 3-Phenyllactic acid | 786 | 0.040 | 0.031 | 1.622 | <0.0001 | 0.768 |
| Nucleosides, Nucleotides, and Analogues | | | | | | |
| thymidine 1 | 687 | 0.048 | 0.058 | 1.426 | 0.0004 | 1.219 |
| Unclassified |  |  |  |  |  |  |
| 3-hydroxybutyric acid | 815 | 0.022 | 0.043 | 1.662 | <0.0001 | 1.928 |
| heptadecanoic acid | 805 | 0.673 | 0.910 | 1.480 | <0.0001 | 1.352 |
| D-Talose 2 | 782 | 0.903 | 1.314 | 1.671 | <0.0001 | 1.456 |
| lactic acid | 683 | 1.429 | 2.263 | 1.644 | <0.0001 | 1.583 |
| methionine sulfoxide 2 | 677 | 0.020 | 0.016 | 1.133 | 0.0199 | 0.811 |
| D-galacturonic acid 2 | 667 | 0.004 | 0.018 | 1.657 | <0.0001 | 4.385 |
| Monostearin | 655 | 0.006 | 0.018 | 1.027 | 0.0038 | 3.190 |
| conduritol b epoxide 2 | 633 | 0.003 | 0.003 | 1.149 | 0.0166 | 0.912 |
| 6-deoxy-D-glucose 1 | 774 | 0.593 | 0.743 | 1.513 | 0.0001 | 1.251 |
| D-(glycerol 1-phosphate) | 727 | 0.005 | 0.009 | 1.642 | <0.0001 | 1.949 |
| Methyl-beta-D-galactopyranoside | 713 | 0.040 | 0.059 | 1.644 | <0.0001 | 1.466 |

^a^ Similarity, The numerical value indicates the matching degree between the substance and the substance in the standard library. The perfect score is 1000. The closer the value is to 1000, the more accurate the qualitative substance.

^b^ VIP (Variable Importance in the Projection) is the weight of difference caused by the difference between the two groups. The value indicates the importance of the variable. The larger the VIP value, the more important it is to discriminate between two sets of variables.

^c^ *P*-VALUE is the student T test result.

^d^ FC (Fold change) is the multiple of difference, FC value in the table is the average value of FMT group / average value of K88+PBS group, FC> 1 means that the differential metabolites are up-regulated in the FMT group, FC <1 means that the metabolites are down-regulated in the FMT group. Differential metabolite screening criteria in this study are VIP > 1, *P*-VALUE < 0.05, Similarity ≥ 600.
